# Retraction: Depression Among Patients With Type 2 Diabetes Mellitus at King Abdulaziz University Hospital (KAUH): A Cross-Sectional Study

**DOI:** 10.7759/cureus.r245

**Published:** 2026-09-16

**Authors:** Bader Al Qusaibi, Hala Mosli, Wid Kattan, Hamza Fadel, Abdulaziz Alariefy, Basim Almalki, Loai Bahkali, Abdulaziz Alzubaidi

**Affiliations:** 1 Department of Medicine, College of Medicine, King Abdulaziz University, Jeddah, SAU; 2 Department of Endocrinology and Metabolism, King Abdulaziz University, Jeddah, SAU; 3 Department of Endocrinology, King Abdulaziz University Hospital, Jeddah, SAU

This article has been retracted by the Editor-in-Chief. Following a reader inquiry, the editorial office reviewed the article's statistical reporting. The odds ratios reported in Tables 3 and 4 cannot be derived from the participant counts presented alongside them, and the article's principal association is reported in opposite directions in the Results and the Discussion. The Abstract and Conclusions rest on these estimates, which are therefore unreliable.

Resolving the discrepancies would require the underlying dataset and regression output. The editorial office contacted all eight listed authors individually on two occasions requesting these materials, and received no response. The findings cannot be verified or corrected.

